# Supplementary material for: Evaluation of Anti-Neuroinflammatory Activity of Isatin Derivatives in Activated Microglia
Source: Molecules. 2023 Jun 20;28(12):4882. doi: 10.3390/molecules28124882 (PMC10301261; doi:10.3390/molecules28124882)

Supplementary Materials

Evaluation of Anti-Neuroinflammatory Activity of Synthetic Isatin Derivatives in Activated Microglia

Alejandro Cenalmor,[1] Elena Pascual,[1] Sergio Gil-Manso,[2] Rafael Correa-Rocha,[2] José Ramón Suárez[3] and Isabel García-Álvarez*[1]

[1]*Facultad de Ciencias Experimentales, Universidad Francisco de Vitoria, 28223 Pozuelo de Alarcón, Madrid, Spain.*
[2]*Laboratory of Immune-Regulation, Gregorio Marañón Health Research Institute (IISGM),28009 Madrid, Spain.*
[3]*Departamento de Química en Ciencias Farmacéuticas, Facultad de Farmacia, Universidad Complutense de Madrid 28040 Madrid, Spain.*

*Corresponding Author For Isabel García-Álvarez, e-mail: isabel.alvarez@ufv.es

Table of Contents:

Page

Table S1. Cell viability and LPS induced NO release in BV2 cells treated with compounds at concentrations 5, 25 and 50 µM 3

Table S2. IL-6 and TNF- α release by LPS activated BV2 cells treated with non cytotoxic isatin derivatives ………………………………………………... 4

Figure S1. ^{1}H-NMR spectrum of compound **10**………………..………………...... 5

Figure S2. ^{1}H-NMR spectrum of compound **1**……………………..…………..... 6

Figure S3. ^{1}H-NMR spectrum of compound **12**……..…….……..………...…… 7

Figure S4. ^{1}H- and ^{13}C-NMR spectra of compound **13**……………………….…... 8

Figure S5. ^{1}H- and ^{13}C-NMR spectra of compound **14**…………………..….…... 9

Figure S6. ^{1}H- and ^{13}C-NMR spectra of compound **15**…….…………...….…... 10

Figure S7. ^{1}H-NMR spectrum of compound **16** ……..……………..………... 11

Figure S8. ^{1}H- and ^{13}C-NMR spectra of compound **17**…………..……………..... 12

Figure S9. ^{1}H- and ^{13}C-NMR spectra of compound **18**……………..……….….. 13

Figure S10. ^{1}H-NMR spectrum of compound **19**…………….…………...…… 14

Figure S11. ^{1}H- and ^{13}C-NMR spectra of compound **20**……………………….....	15

Figure S12. ^{1}H- and ^{13}C-NMR spectra of compound **21**……………………….....	16

Figure S13. ^{1}H- and ^{13}C-NMR spectra of compound **22**……………………….....	17

Figure S14. ^{1}H-NMR spectrum of compound **23**………………………...…......	18

Table S1. Cell viability and LPS induced NO release in BV2 cells treated with compounds at concentrations of 5, 25 and 50 μM.[a]

	Cell viability (%)			[nitrite][b] (uM)		
**Compd.**	**5 μM**	**25 μM**	**50 μM**	**5 μM**	**25 μM**	**50 μM**
**1**	105 ± 1	106 ± 2	103 ± 3	4.19 ± 0.40	5.61 ± 0.40	5.11 ± 0.40
**2**	109 ± 4	107 ± 3	110 ± 4	5.28 ± 0.23	4.25 ± 0.20	4.53 ± 0.20
**3**	105 ± 3	90 ± 3	50 ± 3****	3.52 ± 0.15	2.33 ± 0.30***	N.D.[c]
**4**	107 ± 7	68 ± 3***	39 ± 1***	4.05 ± 0.37	2.71 ± 0.45**	N.D.
**5**	100 ± 4	68 ± 3***	34 ± 3***	3.24 ± 0.12*	2.64 ± 0.04**	N.D.
**6**	101 ± 7	55 ± 2***	28 ± 2****	3.16 ± 0.18*	2.52 ± 0.10**	N.D.
**7**	118 ± 5	115 ± 4	93 ± 5	4.80 ± 0.17	2.92 ± 0.22**	3.04 ± 0.11*
**8**	114 ± 3	120 ± 4	99 ± 4	3.71 ± 1.45	3.52 ± 0.25	3.03 ± 0.18
**9**	105 ± 5	115 ± 3	120 ± 3	4.13 ± 0.98	4.36 ± 0.33	3.71 ± 0.40
**10**	102 ± 2	97 ± 2	80 ± 3	2.27 ± 0.17**	1.70 ± 0.15****	1.60 ± 0.14****
**11**	100 ± 2	100 ± 3	90 ± 2	5.49 ± 0.52	4.24 ± 0.60	3.00 ± 0.43****
**12**	90 ± 3	92 ± 4	84 ± 3	4.11 ± 0.12	2.60 ± 0.12**	1.60 ± 0.05****
**13**	105 ± 2	110 ± 2	114 ± 2	3.91 ± 0.13	3.30 ± 0.20	2.50 ± 0.21*
**14**	108 ± 2	92 ± 1	85 ± 3	4.43 ± 0.30	4.71 ± 0.24	4.05 ± 0.34
**15**	106 ± 1	100 ± 2	102 ± 1	4.00 ± 0.20	3.65 ± 0.13	3.31 ± 0.13
**16**	97 ± 2	85 ± 3*	80 ± 2*	6.72 ± 0.81	7.00 ± 1.00	5.84 ± 0.43
**17**	100 ± 2	67 ± 1***	24 ± 3****	2.65 ± 0.30***	1.16 ± 0.12****	N.D.
**18**	100 ± 3	82 ± 3**	21 ± 2****	3.60 ± 0.30	1.53 ± 0.30****	N.D.
**19**	100 ± 2	85 ± 2*	65 ± 2***	3.90 ± 0.23	1.60 ± 0.07****	N.D.
**20**	100 ± 1	106 ± 2	93 ± 2	2.74 ± 0.12***	1.45 ± 0.12****	1.20 ± 0.20****
**21**	113 ± 4	110 ± 3	100 ± 2	4.72 ± 0.21	4.41 ± 0.30	4.73 ± 0.53*
**22**	100 ± 2	106 ± 3	85 ± 1	3.67 ± 0.30	2.55 ± 0.30***	1.70 ± 0.15***
**23**	95 ± 4	83 ± 3**	70 ± 3**	10.71 ± 0.74*	11.10 ± 0.30*	N.D.

[a] Data are presented as the mean value ± SEM of at least three independent experiments performed in triplicate. Cells were pretreated for 1 h with isatin derivatives (5, 25 or 50 μM) or vehicle (0.025% DMSO) followed by LPS stimulation (1 μg/mL) and incubation for 24 h at 37 °C. [b]NO production was determined by Griess test. [c]N.D. not determined due to low cell viability. LPS stimulated cells treated with vehicle viability was 100 ± 3 % and [nitrite] = 4.52 ± 0.12 μM. *$p < 0.05$, **$p < 0.01$, ***$p < 0.001$, ****$p < 0.0001$ compared to LPS-stimulated cells treated with vehicle.

Table S2. IL-6 and TNF-α release by LPS-activated BV2 cells treated with non cytotoxic isatin derivatives.[a]

Treatment compound	[IL-6] (pg/mL)[b]	[TNF-α] (pg/mL)[b]
Control[c]	260 ± 28****	206 ± 22**
LPS	2772 ± 521	728 ± 60
**1**	4504 ± 1591	514 ± 110
**3**	2197 ± 980	274 ± 63*
**10**	1146 ± 182*	394 ± 25*
**11**	3307 ± 1139	541 ± 162
**12**	2362 ± 872	580 ± 83
**13**	2333 ± 343	713 ± 123
**14**	4121 ± 939	887 ± 120
**15**	2406 ± 438	781 ± 62
**20**	1121 ± 374*	391 ± 34*
**21**	4148 ± 1040	946 ± 135
**22**	2018 ± 193	704 ± 21

[a] Data are presented as the mean value ± SEM of at least three independent experiments performed in triplicate. Cells were pretreated for 1 h with isatin derivatives (25 μM) or vehicle (0.025% DMSO) followed by LPS stimulation (1 μg/mL) and incubation for 24 h at 37 °C. IL-6 and TNF-α production were determined by microfluidic ELISA equipment ELLA. [c]Cells treated with vehicle without LPS-stimulation. *$p < 0.05$, **$p < 0.01$, ****$p < 0.0001$ compared to LPS-stimulated cells treated with vehicle.

^{1}H- and ^{13}C-NMR spectra:

Figure S1. ^{1}H-NMR (300 MHz, $CDCl_3$) spectrum of compound **10**:

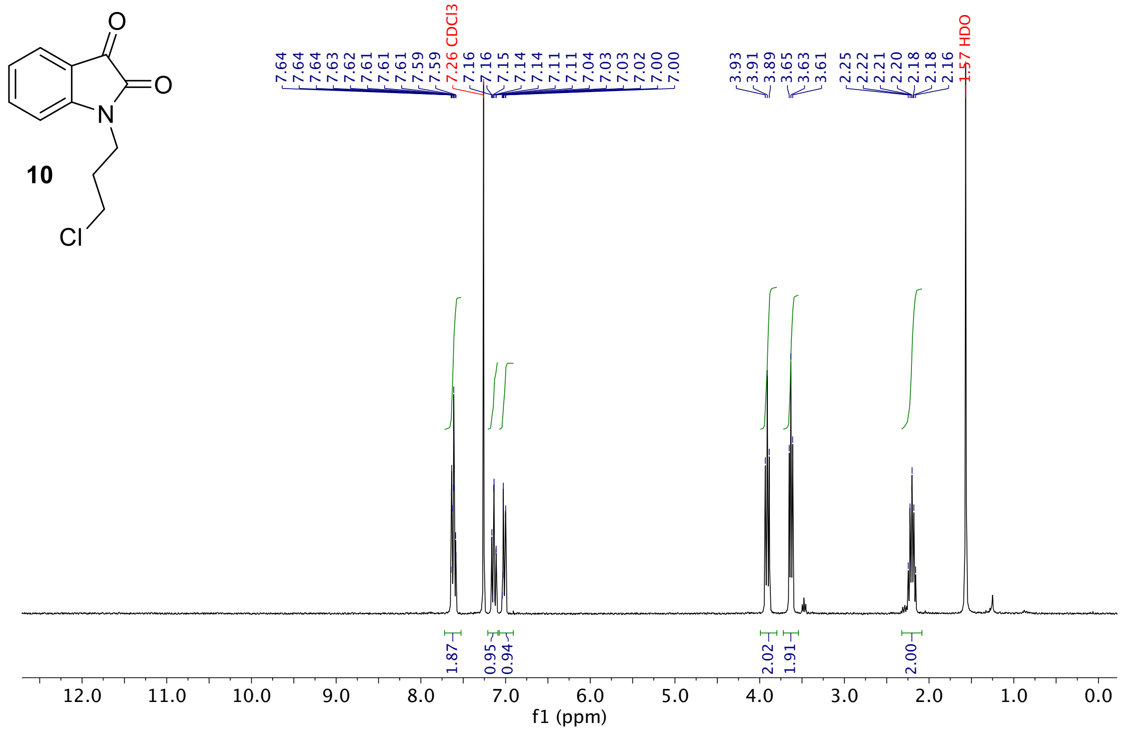

Figure S2. ^{1}H-NMR (300 MHz, $CDCl_3$) spectrum of compound **11:**

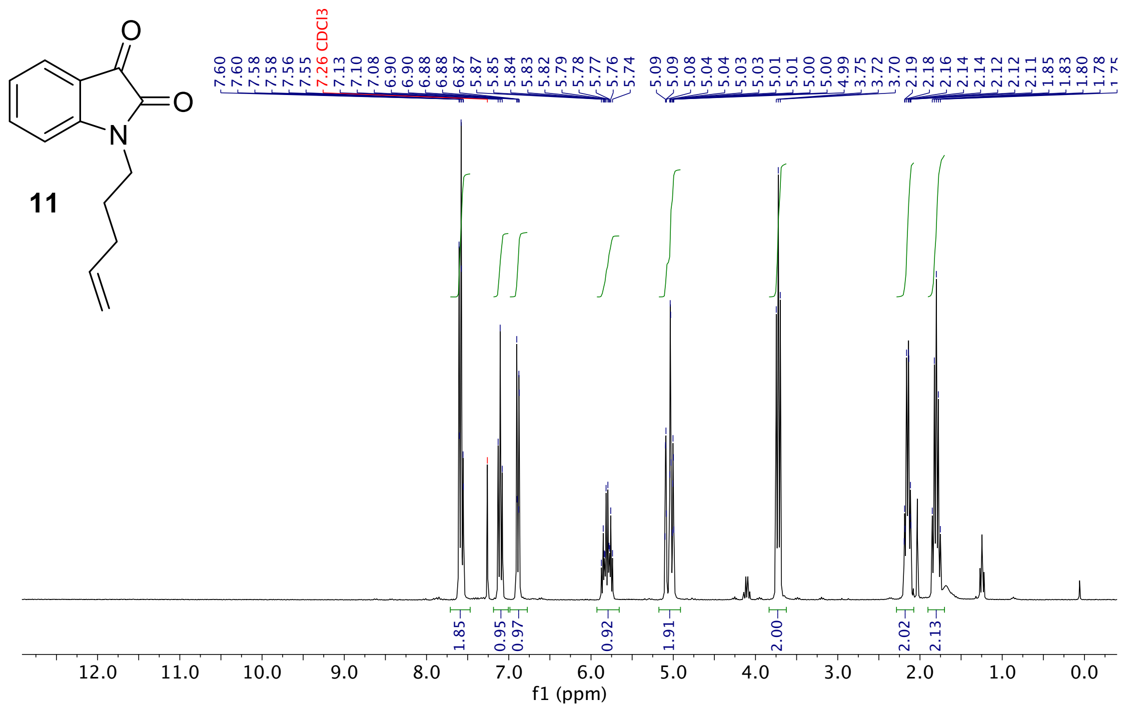

Figure S3. ^{1}H-NMR (300 MHz, $CDCl_3$) spectrum of compound **12:**

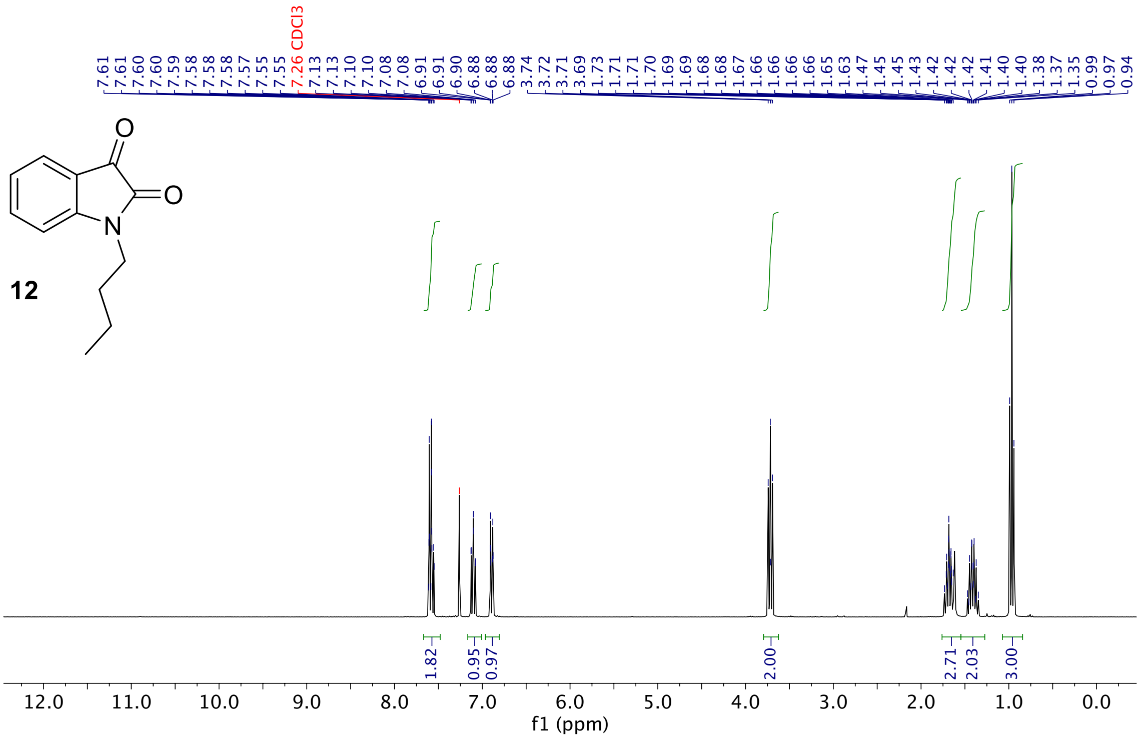

Figure S4. ^{1}H- and ^{13}C-NMR (300 and 75 MHz, DMSO-d_6) spectra of compound **13**:

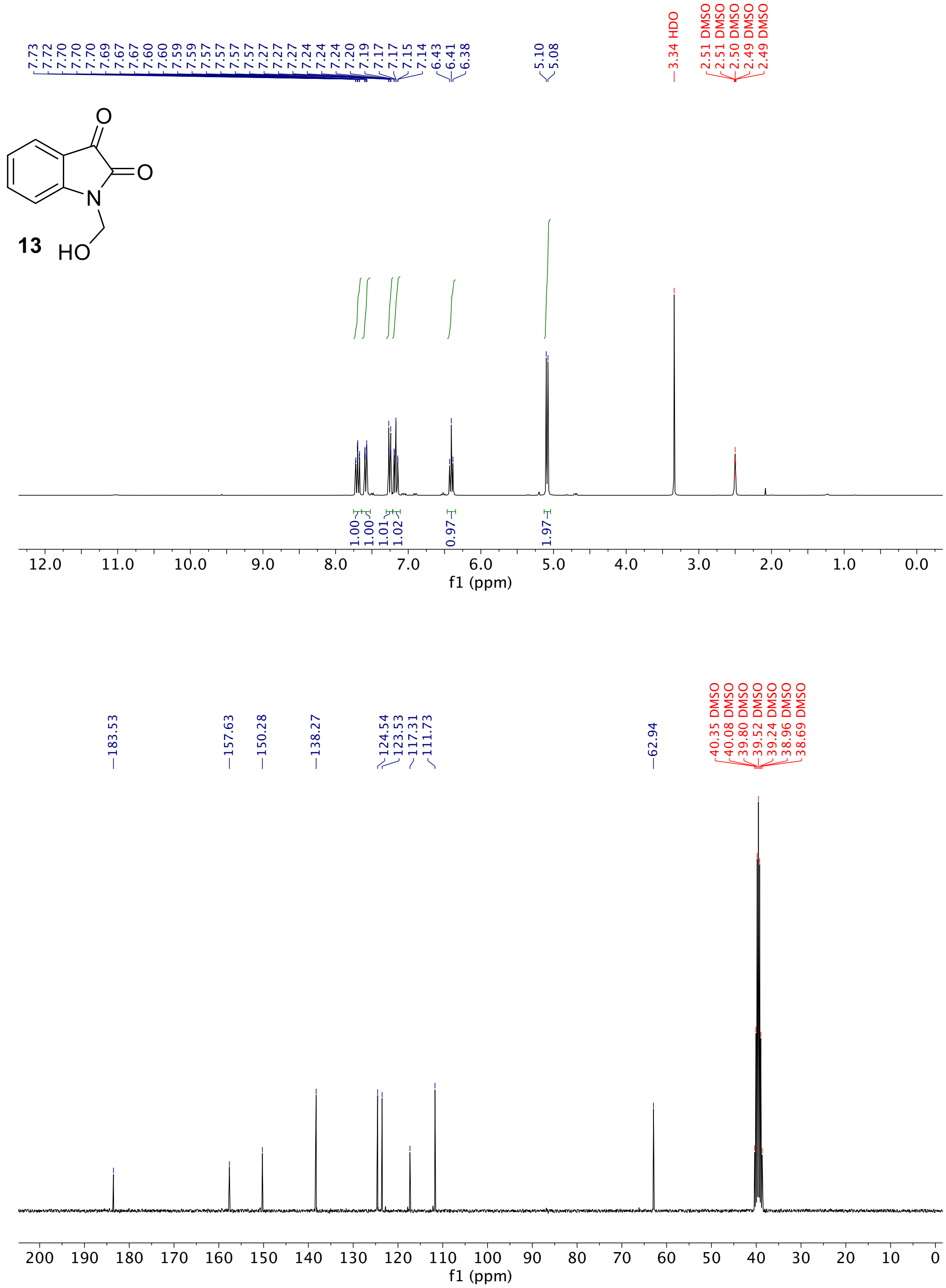

Figure S5. ^{1}H- and ^{13}C-NMR (300 and 75 MHz, DMSO-d_6) spectra of compound **14:**

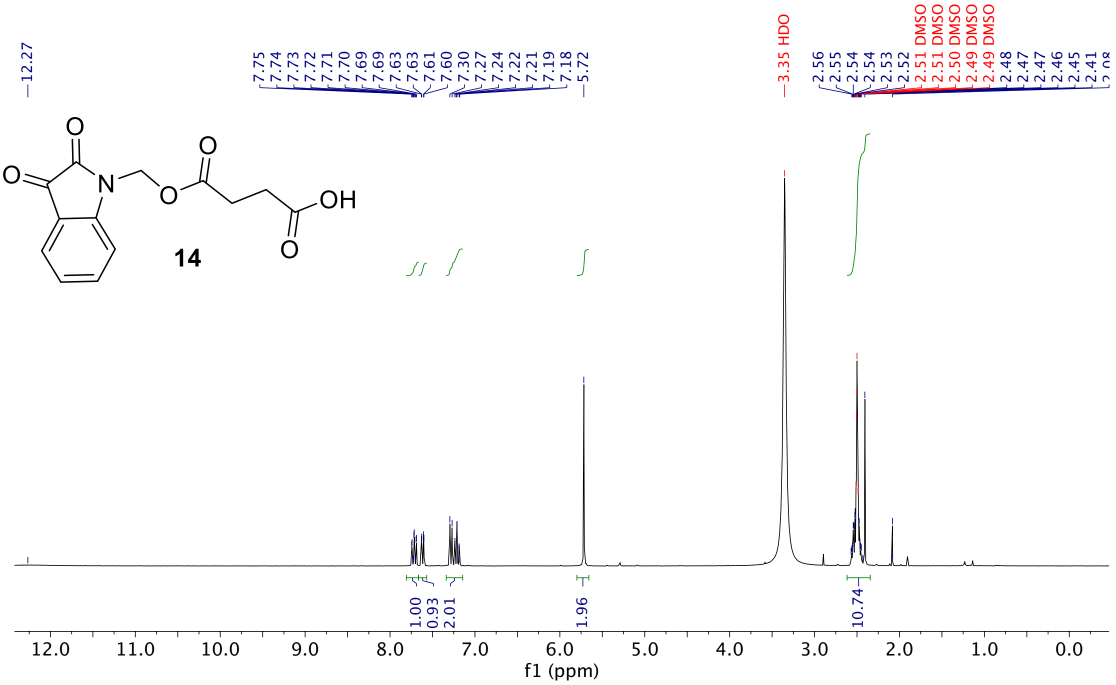

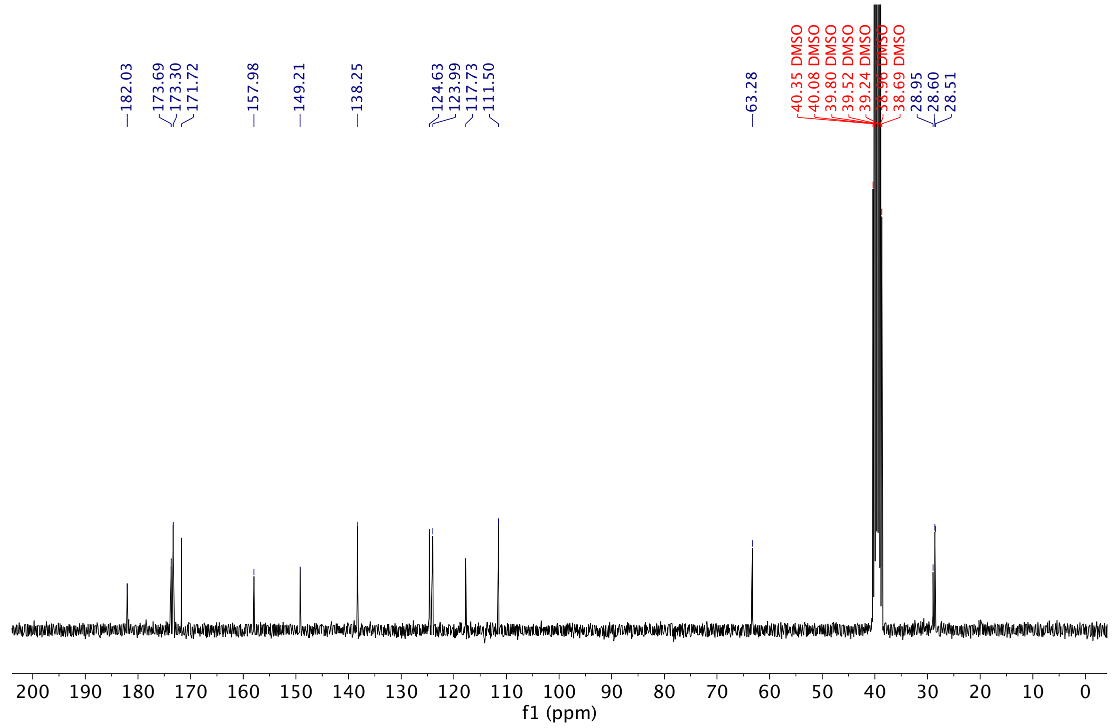

Figure S6. ^{1}H- and ^{13}C-NMR (300 and 75 MHz, DMSO-d_6) spectra of compound **15:**

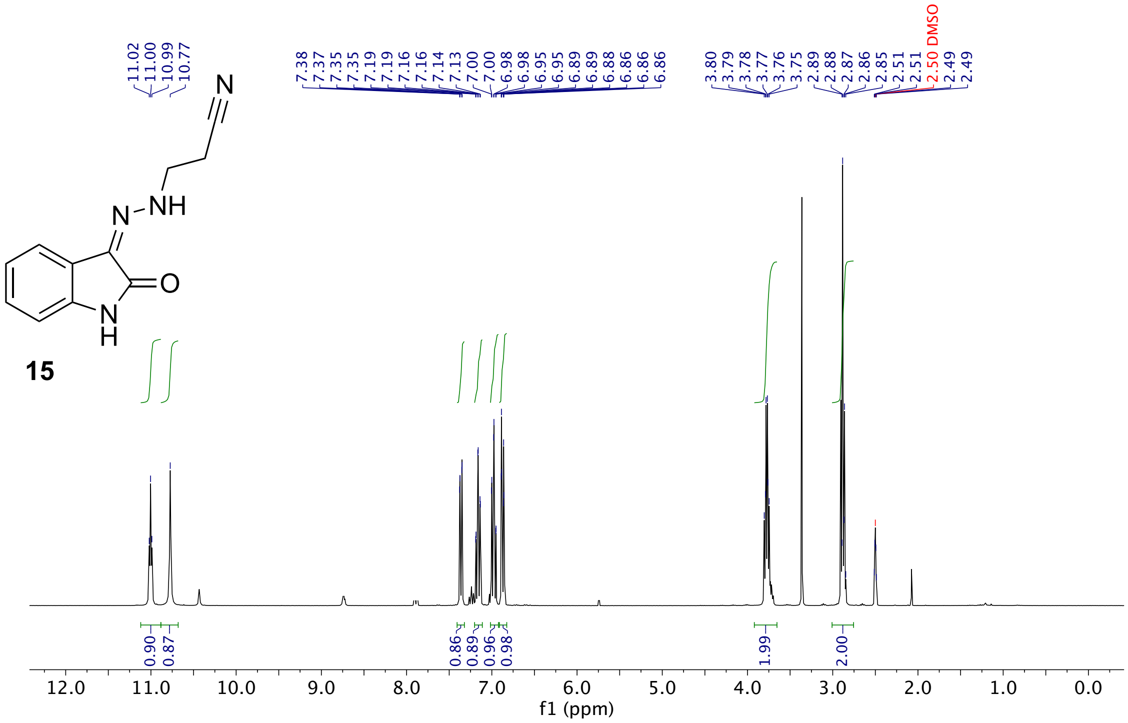

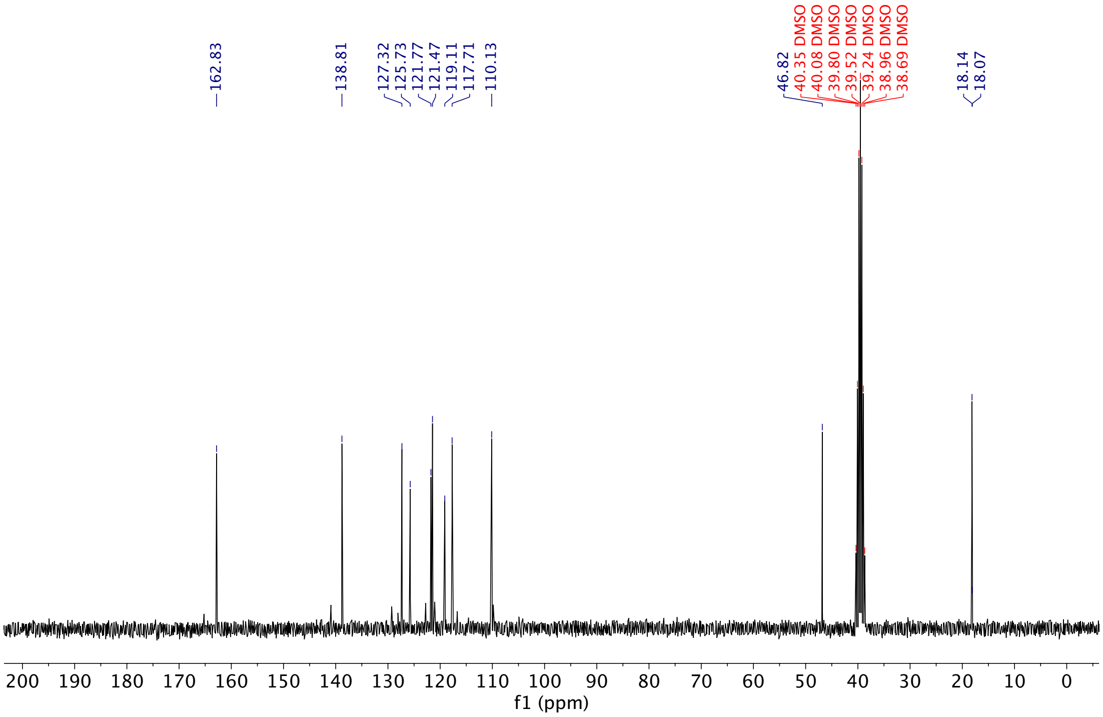

Figure S7. ^{1}H-NMR (300 MHz, DMSO-d_6) spectrum of compound **16:**

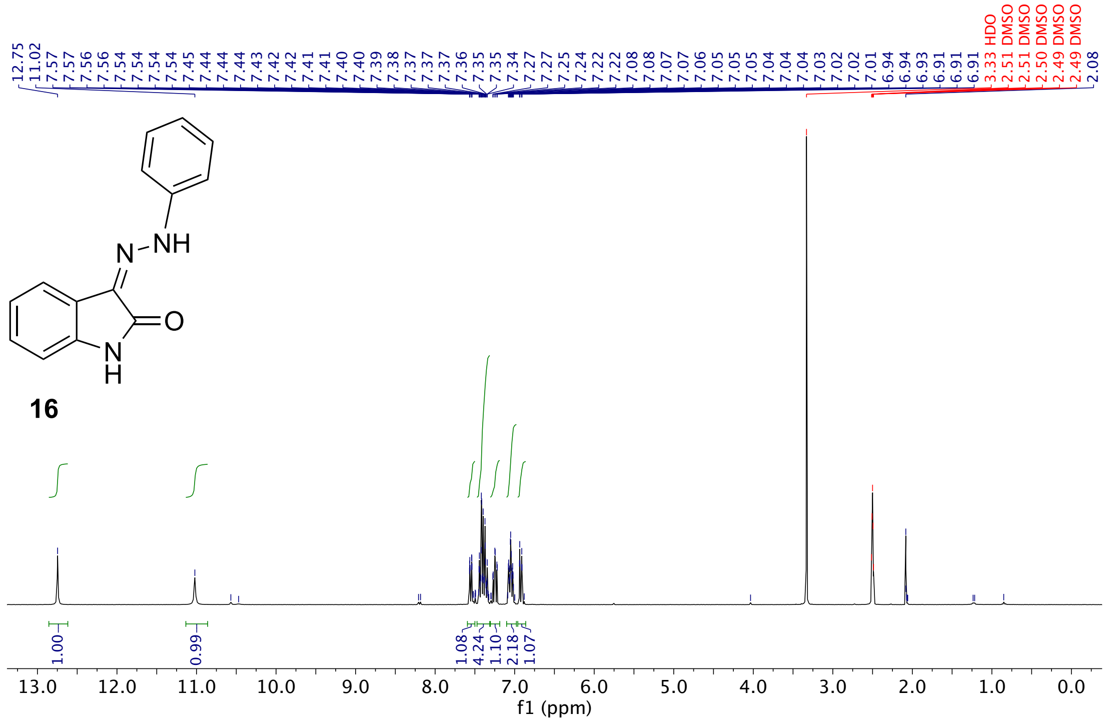

Figure S8. ^{1}H- and ^{13}C-NMR (300 and 75 MHz, $CDCl_3$) spectra of compound **17:**

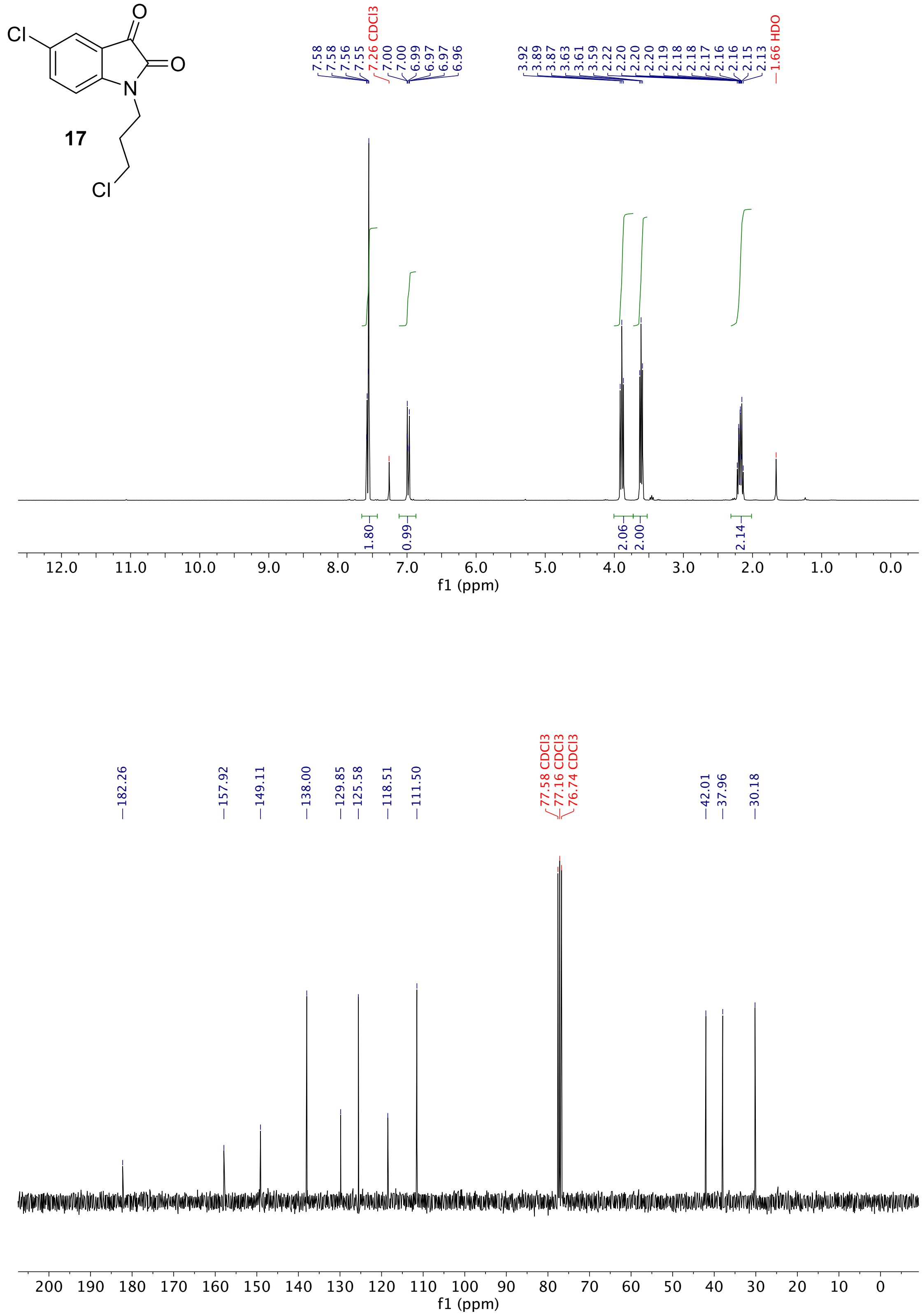

Figure S9. ^{1}H- and ^{13}C-NMR (300 and 75 MHz, $CDCl_3$) spectra of compound **18:**

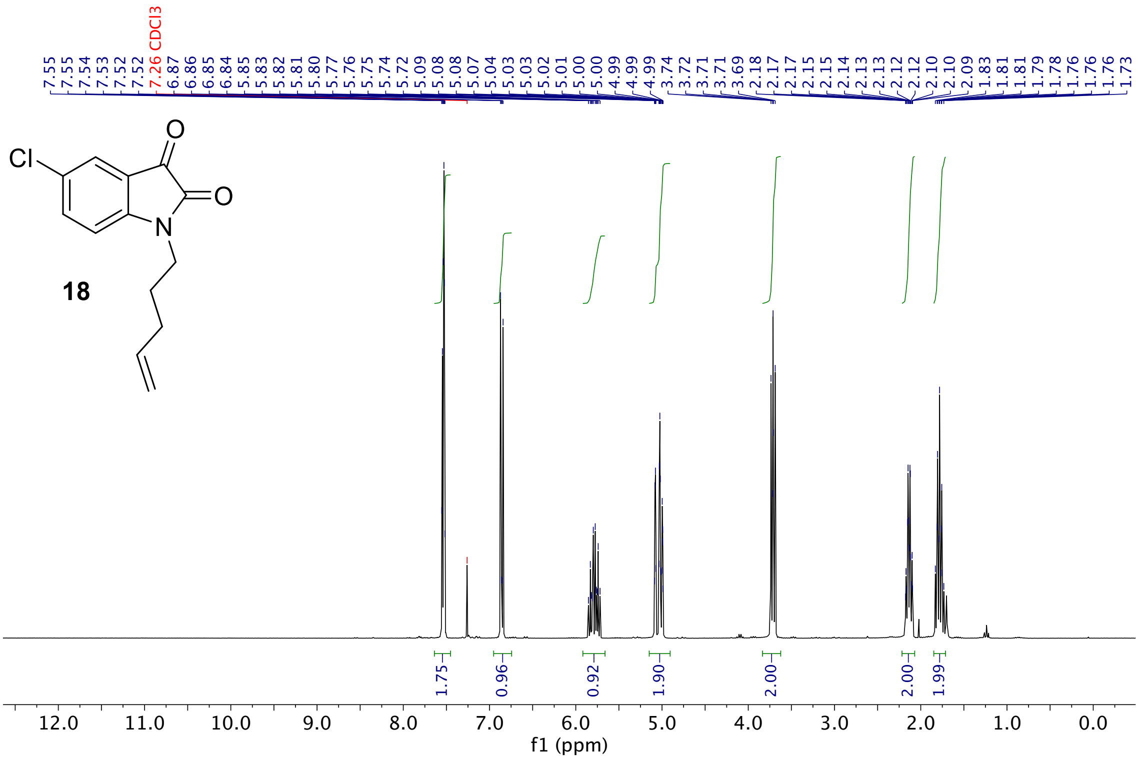

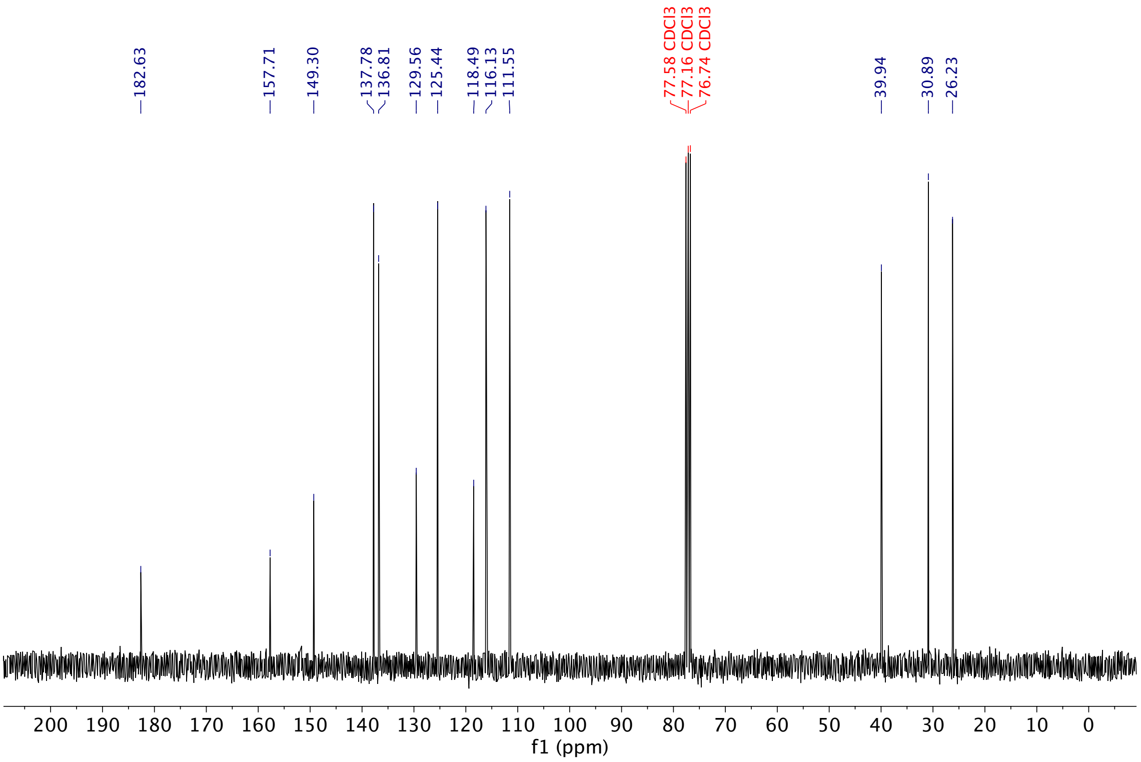

Figure S10. ^{1}H-NMR (300 MHz, $CDCl_3$) spectrum of **19:**

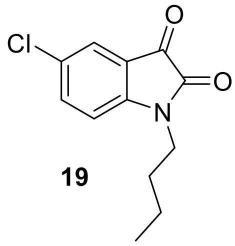

19

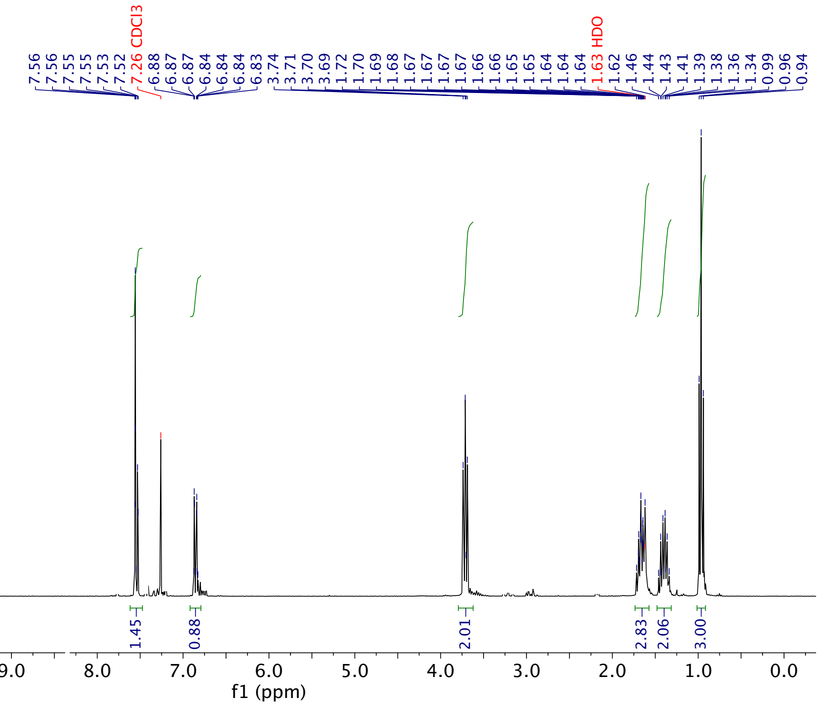

Figure S11. ^{1}H- and ^{13}C-NMR (300 and 75 MHz, DMSO-d_6) spectra of **20:**

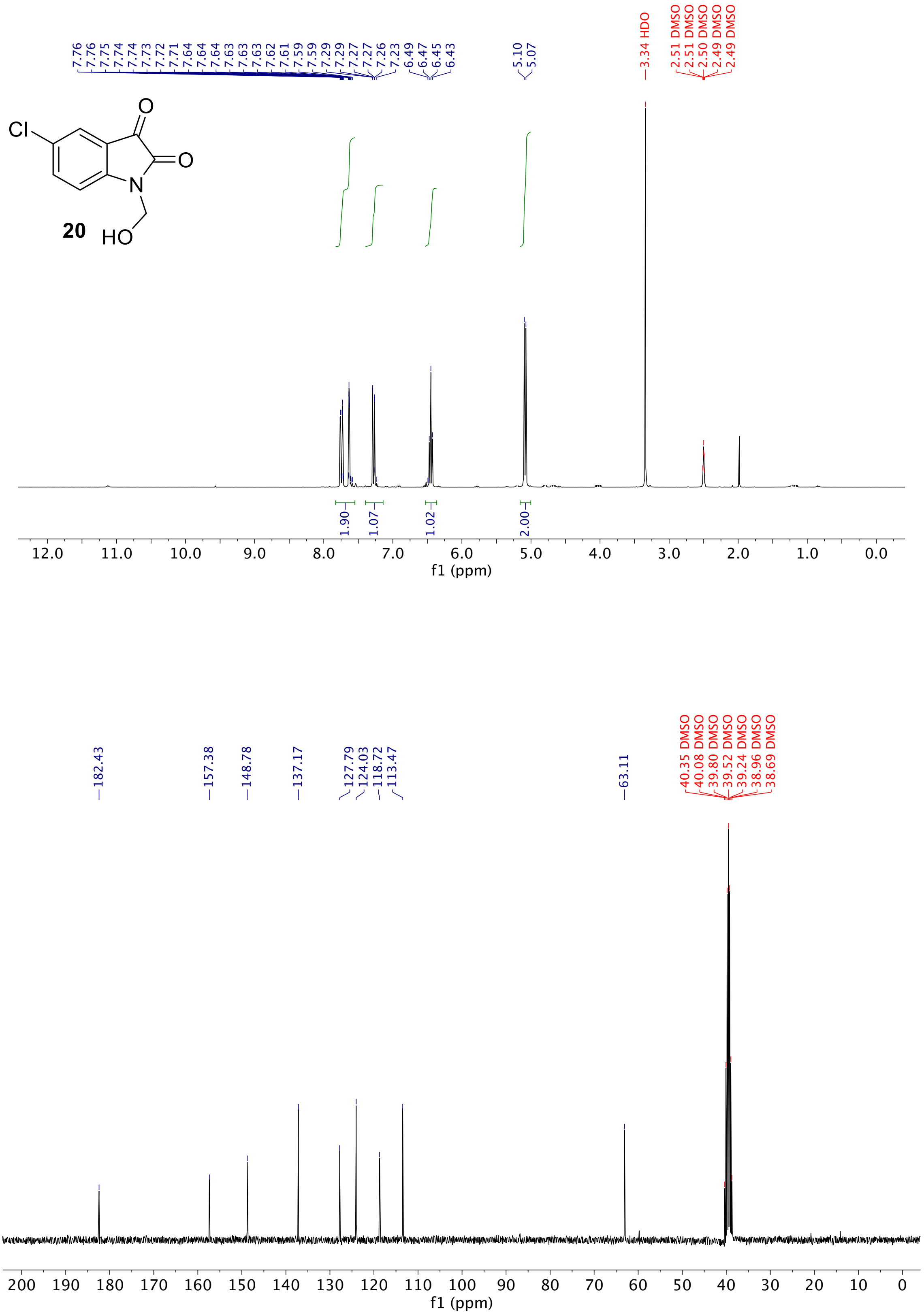

Figure S12. ^{1}H- and ^{13}C-NMR (300 and 75 MHz, DMSO-d_6) spectra of **21**:

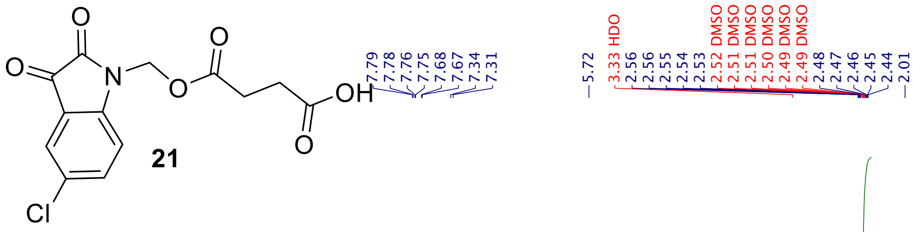

21

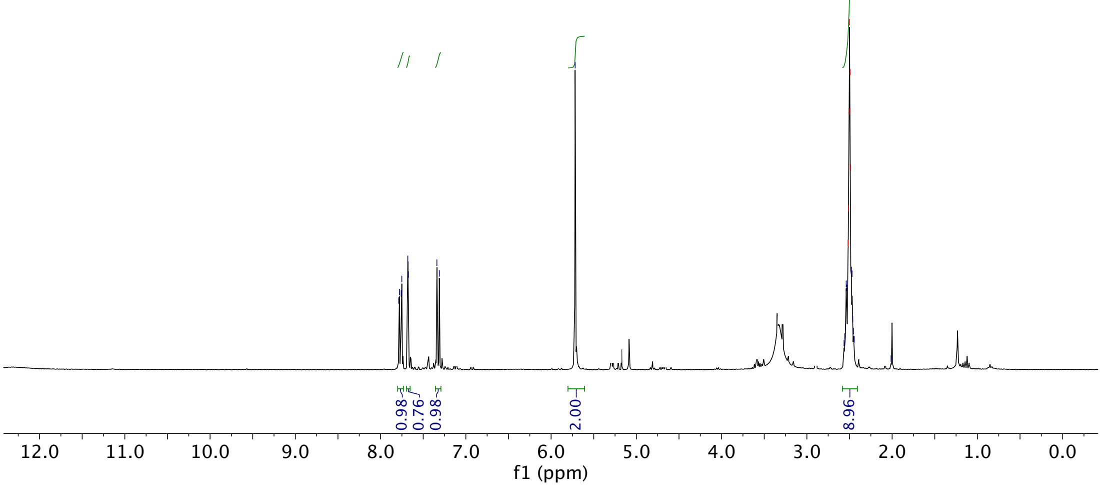

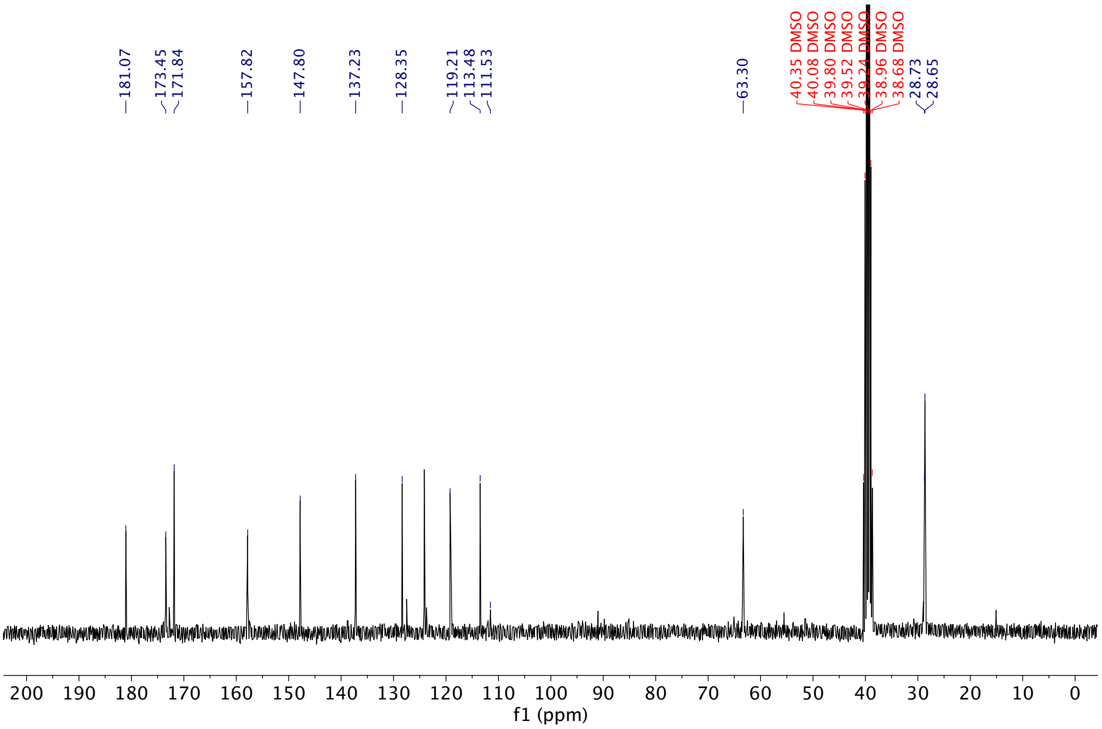

Figure S13. ^{1}H- and ^{13}C-NMR (300 and 75 MHz, DMSO-d_6) spectra of **22:**

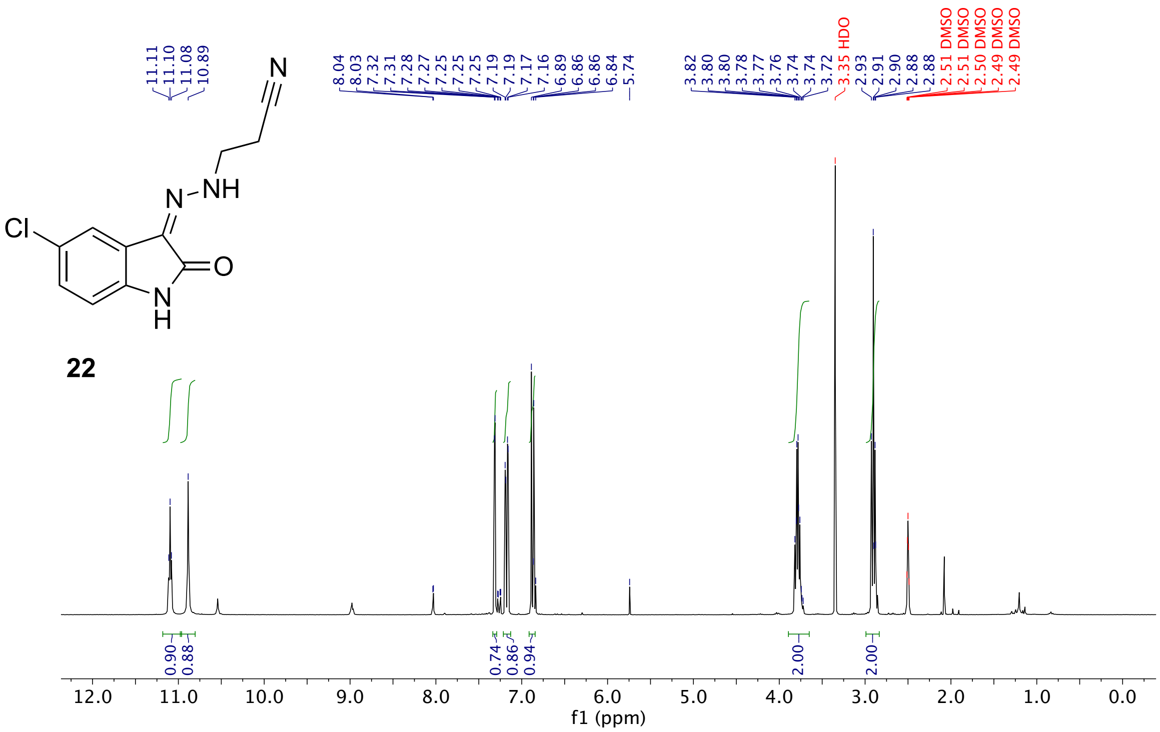

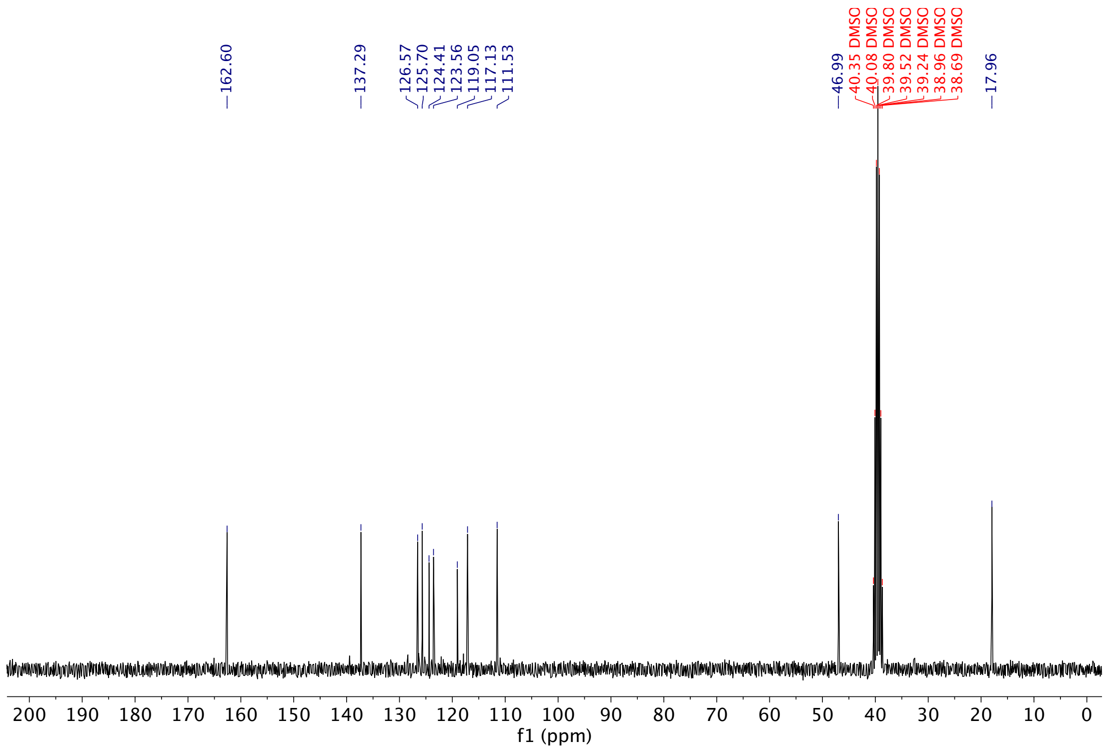

Figure S14. ^{1}H-NMR (300 MHz, DMSO-d_6) spectrum of **23:**

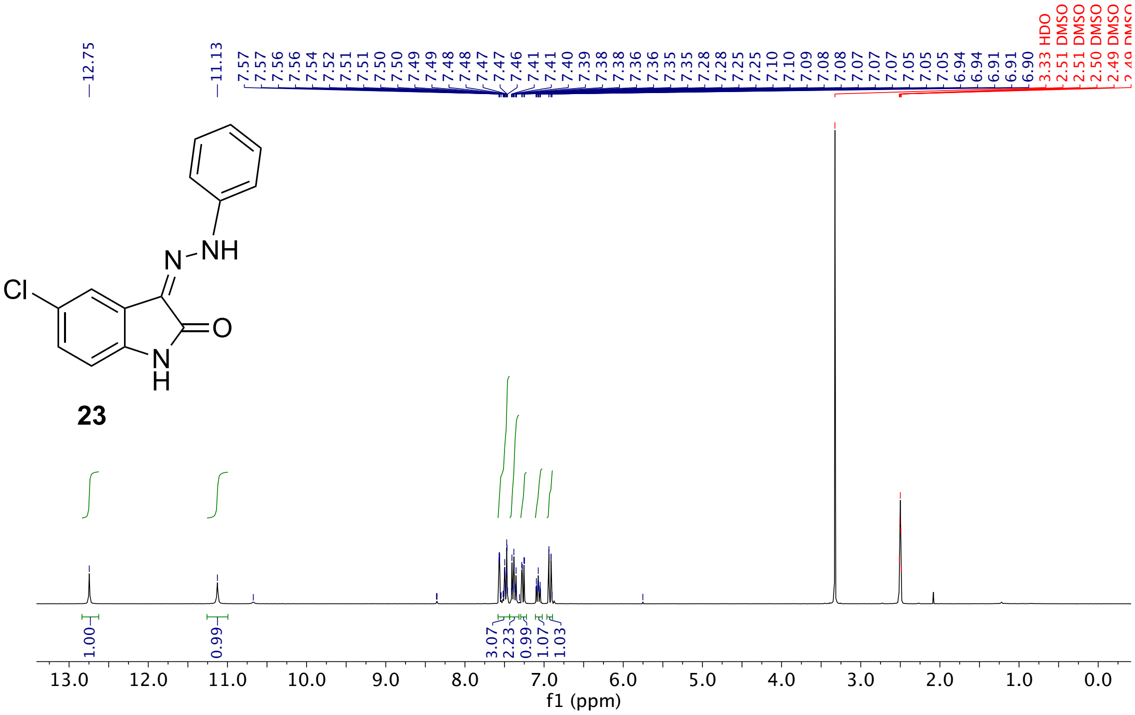

Supplement: Supplementary file 1 [file molecules-28-04882-s001.zip › Supplementary Materials_revised_2.pdf]
